# Behavioral Suppression and Rapid Lethality: *Beauveria bassiana* B4 Targets Adult *Monochamus alternatus* for Sustainable Management of Pine Wilt Disease

**DOI:** 10.3390/insects16101045

**Published:** 2025-10-12

**Authors:** Yaqi Zhang, Xuejie Zhang, Liudi An, Dongfeng Gong, Jinsheng Wang, Huitao Bi, Yi Zheng, Lei Cao, Shaohui Lu

**Affiliations:** 1 College of Forestry, Henan Agricultural University, Zhengzhou 450046, China; zyqttaxx@163.com (Y.Z.); bihuitao@126.com (H.B.); 2 Henan Academy of Forestry, Zhengzhou 450002, China; ald2025662@163.com (L.A.); gongdongfeng@126.com (D.G.); 3 Xixia Forestry Development Service Center, Nanyang 474500, China; zhangxuejie1978@163.com (X.Z.); caolei196511@163.com (L.C.); 4 School of Plant Protection, Anhui Agricultural University, Heifei 230036, China; wjs20020121@163.com; 5 Puyang Forestry Technology Workstation, Puyang 457099, China; zy1983113066@163.com

**Keywords:** *Beauveria bassiana*, forest trials, sustainable forest management, *Monochamus alternatus*, pathogenicity test, slow-release technology

## Abstract

Pine wilt disease, transmitted primarily by *Monochamus alternatus* (Hope, 1842) adults, causes severe ecological and economic losses globally. Conventional chemical controls face challenges of resistance and non-target toxicity. This study identified *Beauveria bassiana* (Bals.-Criv.) Vuill. strain B4 as a high-virulence biocontrol agent against adult *M. alternatus*. Laboratory bioassays compared four strains (B1–B4), with B4 exhibiting rapid lethality (LT_50_ = 6.61 days at 1 × 10^8^ spores/mL) and low median lethal concentration (LC_50_ = 9.63 × 10^5^ spores/mL). Critically, B4 infection induced significant behavioral suppression, including reduced appetite and mobility prior to death. In forest trials, pheromone-enhanced nonwoven fabric bags impregnated with B4 spores reduced trap catches by 66.4% within one month, with effects persisting for over a year without reapplication. The slow-release carrier system enabled continuous spore dissemination and sustained population suppression. These results demonstrate that B4’s dual action—rapid lethality and behavioral disruption—provides an effective, eco-friendly strategy for sustainable pine wilt disease management.

## Simple Summary

Pine wilt disease is a devastating forest disease that kills millions of pine trees worldwide. It is spread by the pine sawyer beetle. While previous efforts have targeted beetle larvae, they live deep inside the wood and are hard to reach. Instead, we focused on controlling the adult beetles that fly freely in the forest. Control of these beetles has relied on chemical insecticides, which can harm the environment. In this study, we tested a natural and environmentally friendly alternative: a fungus that kills insects. We found a specific type, called *Beauveria bassiana* strain B4, that is very effective at quickly killing the adult beetles. We also observed that infected beetles stop eating and moving long before they die, which means they likely stop spreading the disease. To use this in the forest, we created a special bag that slowly releases the fungus over time. When we hung these bags in the forest, they successfully attracted and killed beetles, reducing their numbers by over 66% in one month, and the effect lasted for more than a year. This method provides a sustainable and effective way to protect our forests without harmful chemicals.

## 1. Introduction

*Monochamus alternatus* is the main vector of nematode-carried pine wilt disease, which primarily affects *Pinus massoniana* Lamb., among other conifers [1]. By feeding on or ovipositing in healthy pine phloem, *M. alternatus* transmits the pathogenic pine wood nematode *Bursaphelenchus xylophilus* (Steiner and Buhrer, 1934) Nickle, 1970 to the host xylem, which results in the large-scale wilting or death of pine trees [2]. Pine wilt disease has devastated global pine forest ecosystems, and the direct economic losses caused by pine wood nematodes in China alone exceed billions of yuan per year (equivalent to approximately hundreds of millions of U.S. dollars based on the average exchange rate) [3]. At present, the prevention and control of *M. alternatus* is highly dependent on chemical insecticides (such as thiacloprid and beta-cypermethrin), but problems caused by long-term insecticide use, such as increased resistance, non-target mortality, and environmental pollution, have become increasingly prominent [4,5,6,7]. Therefore, the development of an environmentally friendly and sustainable biological control is urgently needed.

Entomopathogenic fungi are natural regulators of insect populations and have shown unique advantages in the comprehensive management of agricultural and forestry pests [8,9,10,11]. *Beauveria bassiana* is a broad-spectrum entomogenous fungus [12] that can cause insect mortality through surface infection, toxin secretion, and nutrient competition. It has been successfully applied to prevent and control locusts, aphids, *Anastrepha ludens* (Loew), and numerous Coleoptera [13,14,15,16]. Studies have shown that *B. bassiana* has significant pathogenicity to *M. alternatus* larvae, and the corrected mortality of larvae treated with a spore suspension (1 × 10^8^ spores/mL) for 7 days can reach more than 80% [17]. Studies on the use of *B. bassiana* and *Metarhizium anisopliae* (Metschn.) Sorokīn have focused on the prevention and control of *M. alternatus* larvae [18,19,20,21,22]. *M. alternatus* larvae are mostly active in the deeper xylem of pine trees. Even if fungicides have a significant effect in the laboratory, it is difficult to cause substantial damage to the larvae in the xylem of trees. However, *M. alternatus* adults feed and mate on needles and other parts of the tree. An Unmanned Aerial Vehicle can cover a large range and provide a means to control *M. alternatus* adults.

To disrupt the transmission of the pathogenic nematode *B. xylophilus*, the causative agent of pine wilt disease, this study targeted its key vector insect, *M. alternatus*, using the entomopathogenic fungus *B. bassiana*. We first screened high-efficiency *B. bassiana* strains and determined their median lethal concentration (LC_50_) and median lethal time (LT_50_) against *M. alternatus* adults in the laboratory. Subsequently, a forest experiment was designed based on the occurrence period of *M. alternatus* adults to evaluate the control efficacy of the most virulent strain applied with nonwoven fabric. Our findings provide a foundation for the biological control of *M. alternatus* and contribute to optimizing the integrated management of pine wilt disease.

## 2. Materials and Methods

### 2.1. Tested Insects and Fungal Strains

Adult *M. alternatus* were collected from *Pinus massoniana* forest in Xixia County (111°01′–111°46′ E 33°05′–33°48′ N and 181–2212.5 m above sea level), Nanyang City, Henan Province, China. The insects were collected from a BF-8 pine sawyer trap (Hangzhou Feiluomeng Biotechnology Co., Ltd., Hangzhou, China) every 2 days, A total of approximately 1600 individuals were captured. These insects were transported to the laboratory and acclimated for 5 days with fresh pine branches. After this period, healthy and active individuals were selected based on morphological identification, resulting in a final cohort of 1440 insects for the experiment. Four strains of *B. bassiana*, identified as B1, B2, B3, and B4, were provided by Baiyun Biological Company of Jiyuan City, Henan Province. These strains were selected for this study because they displayed varying phenotypic characteristics (e.g., colony morphology, growth rate) in preliminary observations, indicating their potential for differential virulence, which was then empirically tested against *M. alternatus*.

### 2.2. Preparation of Culture Medium and Fungal Suspensions

The *B. bassiana* strains were cultured in PDA medium containing streptomycin sulfate and kanamycin at 25 °C for 7 d. The surface of the colony was then washed with sterile water to obtain a conidial suspension. Conidial suspensions with concentrations of 1.0 × 10^8^, 1.0 × 10^7^, 1.0 × 10^6^, 1.0 × 10^5^, and 1.0 × 10^4^ spores/mL were prepared with 0.05% Tween-80 solution for the inoculation test.

### 2.3. Nonwoven Bags and Attractants

We manufactured nonwoven bags for *B. bassiana* strain B4 and an F8 attractant (Hangzhou Feiluomeng Biotechnology Co., Ltd.).

### 2.4. Preparation and Inoculation of Nonwoven Bags

Nonwoven fabric bags (25 cm × 30 cm × 5 cm) were filled with sponge blocks of the same size, sterilized, and cooled. A conidial suspension with a concentration ≥ 1 × 10^8^ spores/mL was prepared, and the sterilized nonwoven bags and sponges were immersed in the solution for 24 h. The bags were then dried in the shade.

### 2.5. Determination of the pathogenicity of Beauveria bassiana to Monochamus alternatus

Healthy adult *M. alternatus* of similar size were selected as test insects. Each *B. bassiana* strain was assessed at five concentrations with three replicates of 20 insects each (n = 60 per concentration). Conidial suspensions were poured into a clean Petri dishes, and adult *M. alternatus* were allowed to crawl freely in different concentrations of conidial suspension for 20 s. After the surface of the insect’s body was fully contaminated with spore suspension, the insects were placed in an incubator (25 °C ± 1 °C, 65 ± 5% relative humidity). The control group was treated with sterile water containing 0.05% Tween-80 solution. The mortality of *M. alternatus* was recorded every 24 h. When stiff insects appeared, they were removed to observe subsequent mycelial growth.

### 2.6. Forest Test of the B4 Strain Combined with Nonwoven Fabric

**Figure 1:**
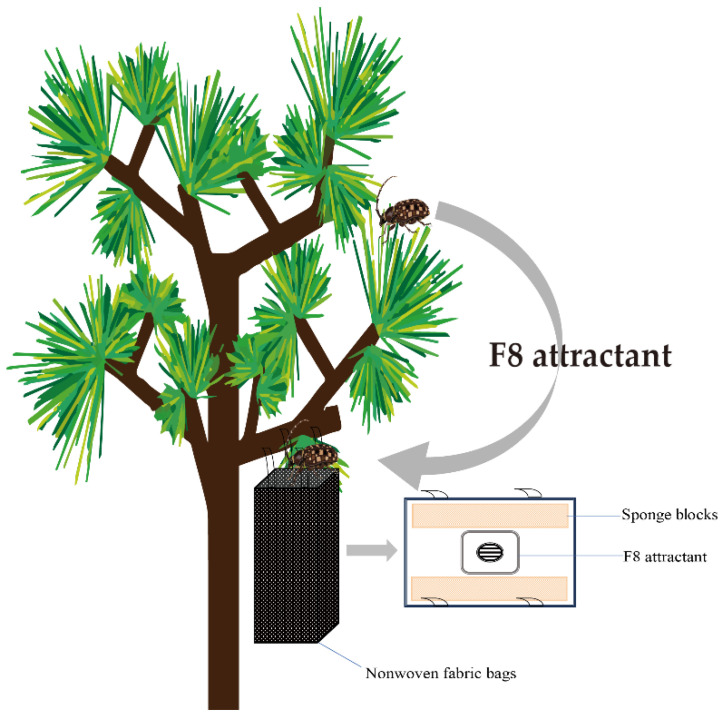
Schematic diagram of the field deployment setup.

The forest experimental site was located in a pine wood nematode epidemic area in Xixia County, Nanyang City, Henan Province (111°28′26″ E 33°18′25″ N and 409 m above sea level). Two 20 ha test plots were selected that contained *P. massoniana* mixed forest. The experiment began at the end of May 2023. The treatment (T1) area was distributed according to a 20 m × 20 m grid, and two nonwoven bags (1.5 m from the ground) were hung at each point. One bottle of F8 attractant was hung 5 cm above the mouth of each bag. A schematic diagram of this field deployment setup is shown in Figure 1. Four traps were hung in the T1 area and the control area at intervals of 50 m. The number of insects in the traps was recorded every 10 d, and the lures were replaced every 2 mo. In 2024, traps were hung at the same locations and the same methods were used to collect data.

### 2.7. Data Analysis

This study included an indoor virulence determination and an outdoor forest prevention and control experiment. In the laboratory, the cumulative mortality and corrected mortality of *M. alternatus* treated with different concentrations of *B. bassiana* spore solution were calculated as follows: η = (μt − μc)/(1 − μc) × 100%, where η is corrected mortality, and the unit is percentage (%); μt is the mortality of the treatment group; and μc is the mortality of the control group. Probit regression analysis was performed using IBM SPSS Statistics (version 26.0, IMB Corp., Armonk, New York, NY, USA) software to establish the virulence equation and calculate LC_50_, LT_50_, and the 95% confidence intervals. The cumulative mortality data, expressed as percentages, were subjected to an arcsine square root transformation to stabilize variances and meet the assumptions of normality and homogeneity of variances for parametric tests. One-way analysis of variance (ANOVA) and Duncan’s multiple range test were then performed on the transformed data. Graphs were made with GraphPad Prism10 (GraphPad Software Inc., Boston, MA, USA) based on the untransformed data for clarity of presentation.

## 3. Results

### 3.1. Screening the Virulence of Beauveria bassiana Strains Following Infection of Monochamus alternatus Adults

At a concentration of 1 × 10^8^ spores/mL, the virulence of the four strains of *B. bassiana* against *M. alternatus* differed significantly (Table 1). The B4 strain was the most pathogenic, and the cumulative corrected mortality rate of *M. alternatus* was 87.18% after 20 d of treatment. The mortality rate induced by the B3 strain was 84.98%. The effects of the B1 and B2 strains were weaker, and the mortality rates were 60.07% and 17.21%, respectively. The virulence of strain B2 was only 19.74% that of B4. The differences among the strains were highly significant, which indicates that genetic background had a decisive influence on the expression of virulence. The above results demonstrated that at the same concentration, *B. bassiana* strains B3 and B4 were the most lethal.

### 3.2. Symptoms of Beauveria bassiana Infection of Monochamus alternatus Adults

**Figure 2:**
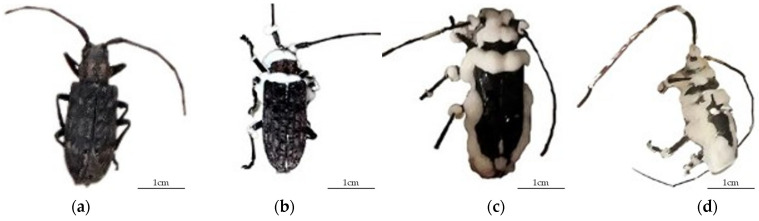
Symptoms of *Beauveria bassiana* B4 infection in adult *Monochamus alternatus.* (**a**) Early infection stage; (**b**) middle infection stage; (**c**) middle-to-late infection stage; (**d**) late infection stage.

In the early stage of infection (1−5 d), *M. alternatus* adults showed symptoms such as decreased appetite and slowness, and mortality soon occurred. In the middle stage of infection (5−10 d), white hyphae began to appear on the surface, particularly around joints and appendages. At the middle-to-late stage of infection (10−15 d), extensive proliferation of mycelia covered large areas of the cuticle. At the late stage of infection (15−25 d), complete coverage of the cadaver by a dense layer of fungal mycelia was observed, producing a powdery layer of white conidia (Figure 2). The progression from exposure to infection, to disease, to death, and to sporulation took a minimum of 5 d and a maximum of 30 d. In most cases, this cycle lasted 10–25 d.

### 3.3. Determining the Toxicity of Strains B3 and B4 to Monochamus alternatus Adults

**Figure 3:**
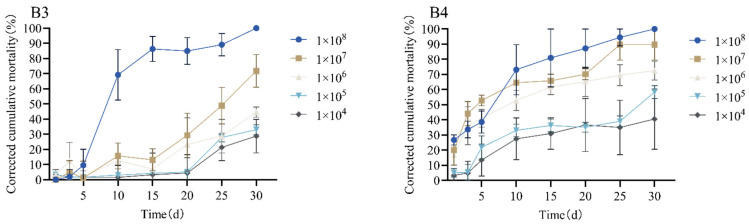
The corrected cumulative mortality of adult *Monochamus alternatus* treated with different concentrations of *Beauveria bassiana* strains B3 and B4.

The virulence of *B. bassiana* strains B3 and B4 against adult *M. alternatus* was evaluated. The LC_50_ values (Table 2) indicate that strain B4 was significantly more virulent than strain B3. Similarly, the LT_50_ and LT_90_ values at different spore concentrations are summarized in Table 3. In general, the LT_50_ values for both strains gradually decreased with increasing spore concentration, and at all tested concentrations, strain B4 exhibited a lower LT_50_ than strain B3, indicating a faster speed of kill. The cumulative corrected mortality over time is shown in Figure 3. For statistical analysis, the mortality percentage data were subjected to an arcsine square root transformation to meet the assumptions of normality prior to ANOVA and DMRT; the figure presents the untransformed means (±SE) for graphical clarity. These results demonstrate the high efficiency of strain B4, even at low doses, supporting its selection for the targeted biological control of *M. alternatus* adults.

### 3.4. Forest Test of Strain B4 Combined with a Nonwoven Bag

The T1 group, in which strain B4 was combined with a nonwoven fabric bag and lure, significantly reduced the number of *M. alternatus* trapped at multiple time points (all *p* < 0.05, Table 4). In the short term (in the first month of 2023), the number of *M. alternatus* trapped in the T1 group (9.5 ± 1.9) was 66.4 % lower than that in the control (CK) group (28.3 ± 4.1) (*p* < 0.001). There was no overlap in the confidence interval (T1: 6.5 ± 12.5; CK: 21.7 ± 34.8), which demonstrates that the treatment effect was stable. In the long term (treatment for 1 year), the number of *M. alternatus* trapped in the T1 group (14.5 ± 2.4) was 30.9 % of that in the CK group (CK: 47.0 ± 2.8); *p* < 0.001). Although *B. Bassiana* spore suspensions were not reapplied in the second year, the number of *M. alternatus* adults trapped in the T1 group (5.75 ± 2.75) was still significantly lower than that in the CK group (37.25 ± 10.08), which indicates that strain B4 had established a stable local population.

## 4. Discussion

The management of mobile insect vectors requires strategies that transcend mere lethal toxicity. Our study demonstrates that the entomopathogenic fungus *B. bassiana* B4 suppresses critical behaviors in adult *M. alternatus* prior to death and that this sublethal effect can be harnessed through an innovative slow-release system to achieve sustained population suppression. This work therefore contributes to the growing field of behavioral manipulation in integrated pest management. Strain B4 showed significant advantages in lethal concentration (LC_50_ = 9.63 × 10^5^ spores/mL) and lethal time (LT_50_ = 6.61 d, the highest concentration treatment), and it was significantly more virulent than strain B3 (LC_50_ = 4.93 × 10^7^ spores/mL, LT_50_ = 11.36 d). This difference in pathogenicity is likely attributable to variations in key physiological characteristics among the strains, such as spore production, germination rate, and cuticle-degrading enzyme activity. Previously, it was shown that the pathogenicity of entomogenous fungi was affected by the combined effects of spore production, the spore germination rate, and body wall penetration ability [23]. In this study, strain B4 caused rapid mortality of *M. alternatus* adults at a low concentration (1 × 10^7^ spores/mL) (LT_50_ = 0.947 d), which demonstrates that its spore germination efficiency and toxin synthesis ability were strong and may have been related to its smaller spore size (2–3 μm) [24]. Small spores are more likely to adhere to the surface of an insect and penetrate the body wall. In addition, the B4 strain could sporulate on the surface of *M. alternatus* adults after infection and form a secondary transmission effect, which increases its potential for continuous prevention and control in a forest environment.

Compared with *M. alternatus* larvae, the prevention and control of *M. alternatus* adults is more challenging. Adults have a wide range of activities and can migrate. Traditional chemical control is difficult to apply accurately and may lead to insecticide resistance. In this study, an indoor toxicity test confirmed that *B. bassiana* had a direct lethal effect on adults, and the cumulative corrected mortality rate reached 90% under a high concentration treatment (1 × 10^8^ spores/mL). Larvae experienced greater than 80% mortality. Previous studies have consistently demonstrated [25,26] that feeding by *Dendrolimus pini* (Linnaeus, 1758) or *M. alternatus* infected with *B. bassiana* decreased sharply (daily food intake decreased by 16.2–16.7%), and their life span was reduced to less than half that of the control group. Although the number of eggs laid by adults was not significantly affected [26], the inhibition of feeding behavior and a shorter lifespan could significantly reduce the transmission of *B. xylophilus*, thus indirectly reducing the spread of pine wilt disease.

A key consideration for the environmental application of entomopathogenic fungi like *B. bassiana* is their potential impact on non-target organisms. A recent study demonstrated that a field-realistic concentration of *B. bassiana* caused increased mortality, altered behavior, reduced reproduction, and induced colony failure in the predatory social wasp *Polistes dominula* (Christ, 1791), a valuable natural enemy of agricultural pests [27]. This inherent challenge underscores the importance of developing targeted application strategies that maximize pest control efficacy while minimizing ecological non-target exposure. In previous studies on the release of biocontrol agents in the forest, nonwoven fabric was bound directly to the trunk of trees, and the biocontrol was not combined with an attractant [28,29]. In this experiment, a nonwoven bag and attractant were combined, and the attractant was used to draw *M. alternatus* adults to the nonwoven bag to become infected by *B. bassiana*, which significantly improved control over *M. alternatus* in the forest. In the treatment with nonwoven bags containing a solution of strain B4, a significantly lower number of *M. alternatus* were trapped; this produced a short-term (within one month) reduction of 66.4%. The prevention and control effect lasted into the next year, which indicates that the B4 strain established a stable population in the forest. Therefore, the targeted nature of our "lure-and-infect" system presents a methodological framework that lends itself to future empirical evaluation of non-target effects under field conditions. Therefore, while the potential for non-target effects exists, our application method is designed to proactively mitigate such risks by limiting environmental dispersal. Future research directly quantifying the impact of this specific strain and application technique on key non-target species in pine forests will be crucial to fully validate its environmental safety and optimize its use in integrated pest management.

It is important to note that the effects of environmental factors on the virulence of *B. bassiana* require further exploration. The laboratory portion of this study was conducted under constant conditions (25 °C, 65% relative humidity), but fluctuations in field temperature and humidity may affect spore germination and infection processes [30,31]. For example, it was found that the LT_50_ of *M. anisopliae* under natural temperature and humidity was extended by 9–15 d, which was significantly different from the laboratory results [21]. It is important to optimize the application of microbial agents for different geographical and climatic conditions, and this may be done by enhancing the environmental tolerance of spores via microcapsule embedding technology [32] or by combining microbial agents with trapping devices to improve targeting.

This study demonstrates the promise of the B4 strain for the biological control of *M. alternatus*; however, several avenues for further research remain to support its practical application. Firstly, while our field trials showed efficacy, the influence of key environmental factors such as ultraviolet radiation and rainfall on fungal persistence was not directly quantified, and potential synergies with other management techniques await exploration [33,34,35]. Secondly, the molecular mechanisms underlying the pathogenicity of strain B4 and the optimization of its large-scale production are crucial areas for future investigation to enhance efficacy and economic viability. Finally, an important consideration for any vector-control strategy is the potential for pathogen transmission during the infection period. Although the short LT_50_ of B4 (6.61 days) likely reduces the window for transmission, future work should directly quantify nematode transmission rates by beetles at different stages of mycosis to fully validate the strategy’s capacity to disrupt the disease cycle.

In summary, we identified *B. bassiana* strain B4 as having high virulence against *M. alternatus*. The use of an attractant and a nonwoven bag in combination with *B. bassiana* strain B4 in the forest was effective, and this method provides a foundation for the biological control of pine wilt disease.

## Figures and Tables

**Table 1:** The cumulative corrected mortality (%) of adult *Monochamus alternatus* caused by different *Beauveria bassiana* strains ^1^.

Days After Inoculation	Fungal Strains			
	B1	B2	B3	B4
1 d	0.00 ± 0.00 b	6.67 ± 11.55 b	0.00 ± 0.00 b	26.67 ± 5.77 a
3 d	32.85 ± 15.00 a	1.95 ± 8.36 b	1.95 ± 8.36 b	33.59 ± 18.70 a
5 d	34.12 ± 12.23 ab	1.18 ± 25.45 b	9.54 ± 18.32 ab	38.56 ± 12.61 a
10 d	40.90 ± 12.19 ab	−1.63 ± 29.30 b	69.19 ± 28.83 a	73.11 ± 28.17 a
15 d	50.00 ± 7.14 ab	7.74 ± 26.75 b	86.31 ± 14.32 a	80.95 ± 32.99 a
20 d	60.07 ± 23.22 a	17.21 ± 10.56 b	84.98 ± 15.40 a	87.18 ± 22.20 a
25 d	66.00 ± 19.15 b	19.56 ± 9.95 c	89.10 ± 12.80 ab	94.44 ± 9.62 a
30 d	71.13 ± 22.25 b	19.56 ± 9.95 c	100 ± 0.00 a	100 ± 0.00 a

^1^ The data show the means ± SE. In each column, the means with the same letter had no significant difference at the 0.05 level according to Duncan’s multiple range test. The concentration was 1.0 × 10^8^ spores/mL.

**Table 2:** LC_50_ of *Beauveria bassiana* strains B3 and B4 against adult *Monochamus alternatus*.

Strain	LC_50_ (spores/mL)	95% FL ^1^ (spores/mL)		Regression Equation	(χ^2^)
		Lower	Upper		
B3	4.93 × 10^7^	7.01 × 10^6^	10^0.323^	y = 0.728x − 5.597	11.574
B4	9.63 × 10^5^	3.18 ×10^5^	2.89 × 10^6^	y = 0.326x − 1.949	0.847

^1^ FL, fiducial limits. Parameters were derived from probit regression analysis.

**Table 3:** Time to lethality for strains B3 and B4 tested against adult *Monochamus alternatus*.

Strain	Spore Concentration (spores/mL)	LT_50_ (d)	LT_90_ (d)	Regression Equation	Correlation Coefficient
B3	1 × 10^8^	11.36	19.93	y = 0.150x − 1.699	0.804
	1 × 10^7^	24.92	39.15	y = 0.900x − 2.243	0.896
	1 × 10^6^	35.23	60.86	y = 0.050x − 1.762	0.724
	1 × 10^5^	37.76	57.91	y = 0.064x − 2.401	0.799
	1 × 10^4^	40.95	62.41	y = 0.060x − 2.444	0.750
B4	1 × 10^8^	6.61	19.67	y = 0.980x − 0.649	0.965
	1 × 10^7^	7.47	28.64	y = 0.061x − 0.452	0.879
	1 × 10^6^	12.40	41.32	y = 0.044x − 0.549	0.833
	1 × 10^5^	26.28	52.98	y = 0.048x − 1.262	0.786
	1 × 10^4^	31.33	60.64	y = 0.044x − 1.370	0.785

**Table 4:** Comparison of trap catches between the *Beauveria bassiana*-treated group and control group at different time points.

Study Year	Time Point	Treatment Group	Mean ± SD	95% FL ^1^ (mg/mL)		*p*-Value
				Lower	Upper	
2023	30 d	T1	9.5 ± 1.9	6.5	12.5	*p* < 0.001
	-	CK	28.3 ± 4.1	21.7	34.8	-
	60 d	T1	5.0 ± 2.4	1.1	8.9	*p* < 0.001
	-	CK	18.8 ± 3.2	13.7	23.8	-
	One year	T1	14.5 ± 2.4	10.7	18.3	*p* < 0.001
	-	CK	47.0 ± 2.8	42.5	51.5	-
2024	30 d	T1	3.3 ± 2.8	−1.1	7.6	0.002
	-	CK	22.5 ± 7.0	11.3	33.7	-
	60 d	T1	2.5 ± 0.6	1.6	3.4	0.002
	-	CK	14.8 ± 4.6	7.5	22.0	-
	One year	T1	5.75 ± 2.75	1.37	10.13	*p* < 0.001
	-	CK	37.3 ± 10.1	21.21	53.29	-

^1^ FL, fiducial limits. Parameters were derived from probit regression analysis.

## Data Availability

The raw data supporting the conclusions of this article will be made available by the authors upon request.

## Abbreviations

The following abbreviations are used in this manuscript:

ANOVA	Analysis of variance
PDA	Potato Dextrose Agar
RH	Relative Humidity
SE	Standard Error
DMRT	Duncan’s Multiple Range Test
